# Genetic characterization of human enterovirus A71 genotypes C4 and B5 Circulating in Qingdao City, Shandong province, China, from 2023 to 2024

**DOI:** 10.3389/fcimb.2025.1684067

**Published:** 2025-12-16

**Authors:** Jinling Gong, Sitong Liu, Song Liu, Rui Sun, Siying Xiao, Zhilei Su, Xin Jiang, Qiuge Zhang, Xiaoyan Shi, Xianming Liu, Zhaoguo Wang

**Affiliations:** 1 Qingdao Municipal Center for Disease Control and Prevention, Qingdao, Shandong, China; 2 Department of Epidemiology and Health Statistics, The College of Public Health of Qingdao University, Qingdao, China; 3 Department of Neurosurgery, Qingdao Municipal Hospital, Qingdao, China

**Keywords:** hand foot and mouth disease, Enterovirus A71, phylogenetics, variation, recombination

## Abstract

Enterovirus A71 (EV-A71) is one of the main pathogens causing hand, foot, and mouth disease (HFMD), and this virus exhibits substantial genetic diversity. To clarify its genomic evolutionary features, this study conducted a phylodynamic analysis on EV-A71 viruses collected in Qingdao (a northern Chinese city) between 2023 and 2024. EV-A71 was identified using a commercial real-time quantitative PCR (qPCR) assay; EV-A71-positive samples were then inoculated into rhabdomyosarcoma (RD) cells for virus isolation. The complete genome sequences and VP1 gene sequences of EV-A71 strains were amplified and analyzed, with IQ-TREE, SimPlot, and RDP4 software used to evaluate their evolutionary characteristics. Among 2,083 clinical samples, 27 were EV-A71-positive, with 19 isolates successfully cultured. Whole-genome analysis confirmed the co-circulation of EV-A71 genotypes C4 (5 strains) and B5 (14 strains). The C4 strains showed high homology to a strain isolated in China in 2019 and carried six lineage-specific mutations. In contrast, the B5 strains clustered into two distinct lineages, including recombinants that had undergone genetic recombination with coxsackievirus A4 (CV-A4) and coxsackievirus A2 (CV-A2). Notably, all EV-A71 strains collected from Qingdao maintained a serine (S) at the 17th amino acid residue of the VP1 region. This work enhances our understanding of the geographical distribution of EV-A71 by confirming the sustained circulation of genotype B5 in northern China and identifying a novel C4/B5 co-circulation pattern in Qingdao—a pattern reflecting complex local evolutionary dynamics. It emphasizes expanding genomic sequencing coverage to monitor B5 and recombinant strains, refining surveillance, and guiding HFMD prevention and control.

## Introduction

Enterovirus A71 (EV-A71), the primary causative agent of hand, foot, and mouth disease (HFMD), belongs to the Enterovirus genus within the Picornaviridae family. This pathogen exhibits significant genetic diversity. Based on the VP1 region sequence, EV-A71 is classified into seven distinct genotypes (A–G) (Kinobe et al., 2022). Surveillance data indicate distinct spatiotemporal distribution patterns. While EV-A71 B5 genotype has been implicated in recurrent epidemics across the Asia-Pacific region (Wu et al., 2013; Puenpa et al., 2019; Xu et al., 2021; Chau et al., 2024), mainland China has maintained endemic transmission of C4 genotype since 1998 (Yang et al., 2017; Puenpa et al., 2019; Fu et al., 2020; Sun et al., 2020; Xu et al., 2021; Ma et al., 2023). Pre-2023 surveillance documented limited detection of EV-A71 B5 genotype in southern China (Xiamen, Chongqing, and Kunming), predominantly associated with potential travel-related introductions (Yang et al., 2016; Huang et al., 2020; Chen et al., 2021). The 2023 Vietnamese outbreak was characterized by EV-A71 B5 variants featuring a VP1 S17G substitution, which may enhance neurovirulence (Chau et al., 2024). This outbreak underscores EV-A71’s ongoing adaptive evolution and highlights the critical need for continuous genomic surveillance of circulating strains.

This study conducted phylodynamic analysis to characterize the genomic evolution of EV-A71 viruses collected from 2023 to 2024 in Qingdao, a city in northern China. Through comprehensive molecular investigation, we address critical knowledge gaps regarding HFMD transmission networks in northern China and highlight emerging challenges for HFMD outbreak prevention and control.

## Materials and methods

### Sample collection, virus detection and epidemiological data analysis

To minimize sampling bias and ensure standardized testing procedures, specimen collection and laboratory analyses were performed in accordance with the National Guidelines for Prevention and Control of Hand, Foot, and Mouth Disease (2009 Edition). Briefly, during the annual HFMD epidemic season from May to September, 15 to 20 throat swab samples were collected monthly from clinically diagnosed HFMD cases in each of the 10 districts of Qingdao City, using a random sampling approach for surveillance purposes.

Throat swab samples from clinically diagnosed cases of HFMD were collected, stored in a dedicated Universal Transport Medium (UTM) (Yocon, Beijing, China) at 4°C, and transported to the laboratory. Viral RNA was extracted from 200 μL aliquots of clinical specimens using the MagNA Pure 96 DNA and Viral NA Small Volume Kit (Roche Diagnostics, Germany) on the MagNA Pure 96 automated extraction system (Roche Diagnostics, Germany), following the manufacturer’s instructions. Pan-Enterovirus and Enterovirus A71 (EV-A71) were detected respectively using commercial real-time quantitative PCR assays: the Universal Enterovirus Nucleic Acid Detection Kit (Daan Gene, Guangzhou, China) and the EV-A71 Nucleic Acid Detection Kit (Daan Gene, Guangzhou, China) on the QuantStudio 7500 Real-Time PCR System (Applied Biosystems, USA).

We conducted a retrospective analysis of HFMD data collected during the HFMD epidemic seasons (May–September) in 2023 and 2024. Epidemiological data, including age and gender distribution, enterovirus positive rate, EV-A71 proportion, and cluster outbreak characteristics were extracted from the HFMD database maintained by the Qingdao Center for Disease Control and Prevention (CDC). Additionally, records of disease severity grades were retrieved from hospital Case Report Forms (CRFs). Data analysis was performed using descriptive statistics, comparative tests, and aggregation analysis.

### Virus isolation and sequencing

EV-A71-positive samples were inoculated into rhabdomyosarcoma (RD) cells (ATCC CCL-136) for virus isolation. Subsequently, RNA was extracted from the supernatants of PCR-positive RD cell cultures and subjected to targeted capture using a commercial enterovirus capture kit (BAIYITECH, Hangzhou, China). The captured and amplified DNA fragments were converted into Illumina-compatible sequencing libraries using the IMAP Library Construction Kit (Illumina, USA). Paired-end sequencing (2 × 150 bp) was performed on an Illumina NextSeq 2000 platform using a 300-cycle high-output flow cell. Raw reads were quality-trimmed (Q-score ≥30) and adapter sequences were removed using Trimmomatic (v0.39). *De novo* assembly was then performed using MEGAHIT (v1.2.9, https://github.com/voutcn/megahit). Sequence identity was determined using BLAST searches (http://www.ncbi.nlm.nih.gov/BLAST) against the National Center for Biotechnology Information GenBank database (http://www.ncbi.nlm.nih.gov/GenBank).

### Molecular evolutionary analysis

Viral genome characterization was performed through parallel analyses of complete VP1 sequences and whole-genome datasets. Multiple sequence alignment was conducted using the MAFFT online platform (version 7.520) with default parameters (Katoh and Standley, 2013). Maximum likelihood phylogenetic reconstruction was performed using IQ-TREE (Nguyen et al., 2015). The optimal substitution model was determined with ModelFinder (Kalyaanamoorthy et al., 2017), and node support was assessed through 1,000 ultrafast bootstrap replicates. The resulting phylogenetic trees were annotated and visualized using the TVBOT platform (https://www.chiplot.online/tvbot.html) for phylogenetic tree beautification (Xie et al., 2023). Genotype classification required >70% bootstrap support for monophyletic clustering with reference strains.

### Recombination analysis

Given the substantial number of EV-A genome sequences available in the GenBank database, closely related sequences were initially identified using the online BLAST tool. Since recombination events frequently occur in non-structural coding regions, the P2 and P3 regions of the EV-A71 sequences in this study were individually analyzed via BLAST. The sequences exhibiting the highest similarity were subsequently selected for recombination analysis.

Putative recombination events were systematically investigated using a dual-method approach. Primary screening was performed with SimPlot (version 3.5.1) (Lole et al., 1999) using sliding window analysis (200-nucleotide window, 20-nucleotide step size) to visualize nucleotide similarity profiles across full-length genomes. Validation analyses were conducted using RDP4 (version 4.101) (Martin et al., 2015), which incorporates seven algorithms: RDP, GENECONV, BootScan, MaxChi, Chimaera, SiScan, and 3SEQ. Recombination events identified with statistically significant support (p<0.05) by at least three distinct algorithms were considered credible. Reference sequences representing all established EV-A71 genotypes were retrieved from the GenBank database for comparative alignment.

## Results

### Epidemiological profile

In this study, a total of 1,167 HFMD samples were tested during 2023, yielding 929 positive cases (positive rate: 79.6%). During 2024, 916 HFMD samples were analyzed, with 737 positive cases identified (positive rate: 80.5%). The difference in positive rates between the two years was not statistically significant (χ² = 0.392, df = 1, P > 0.5). The EV-A71 detection rate increased from 0.17% (2/1,167) in 2023 to 2.73% (25/916) in 2024. Retrospective epidemiological analyses demonstrated no statistically significant differences in age and gender distributions or in enterovirus positive rate among HFMD cases included in the study between 2023 and 2024 (**Supplementary Tables S1**–**S5**). In addition, the EV-A71-positive cases were distributed across 10 districts with heterogeneous onset times, and no clustered outbreaks associated with EV-A71 were identified. A review of CRFs confirmed that none of the EV-A71-infected cases developed severe clinical manifestations.

Furthermore, we performed a retrospective analysis of the HFMD pathogen spectrum from 2013 to 2024. The results showed that, except in 2017, the proportion of EV-A71 showed a general downward trend over this period, while the proportion of other enterovirus types displayed an overall upward trend (**Supplementary Figure S1**).

### Virus isolation and molecular evolutionary analysis

#### Virus isolation

Virus isolation using RD cell lines demonstrated that 19 EV-A71 strains were cultivable: 1 isolate was successfully recovered from the 2023 collection and 18 from the 2024 collection.

#### Whole genome sequencing and phylogenetic analysis

Whole-genome sequencing identified nineteen EV-A71 strains: five C4 genotype strains (all from 2024) and fourteen B5 genotype strains (one from 2023, thirteen from 2024). Phylogenetic analysis employed complementary approaches: maximum-likelihood reconstruction based on complete genome sequences, combined with targeted analysis of the complete VP1 coding regions.

**Figure 1:**
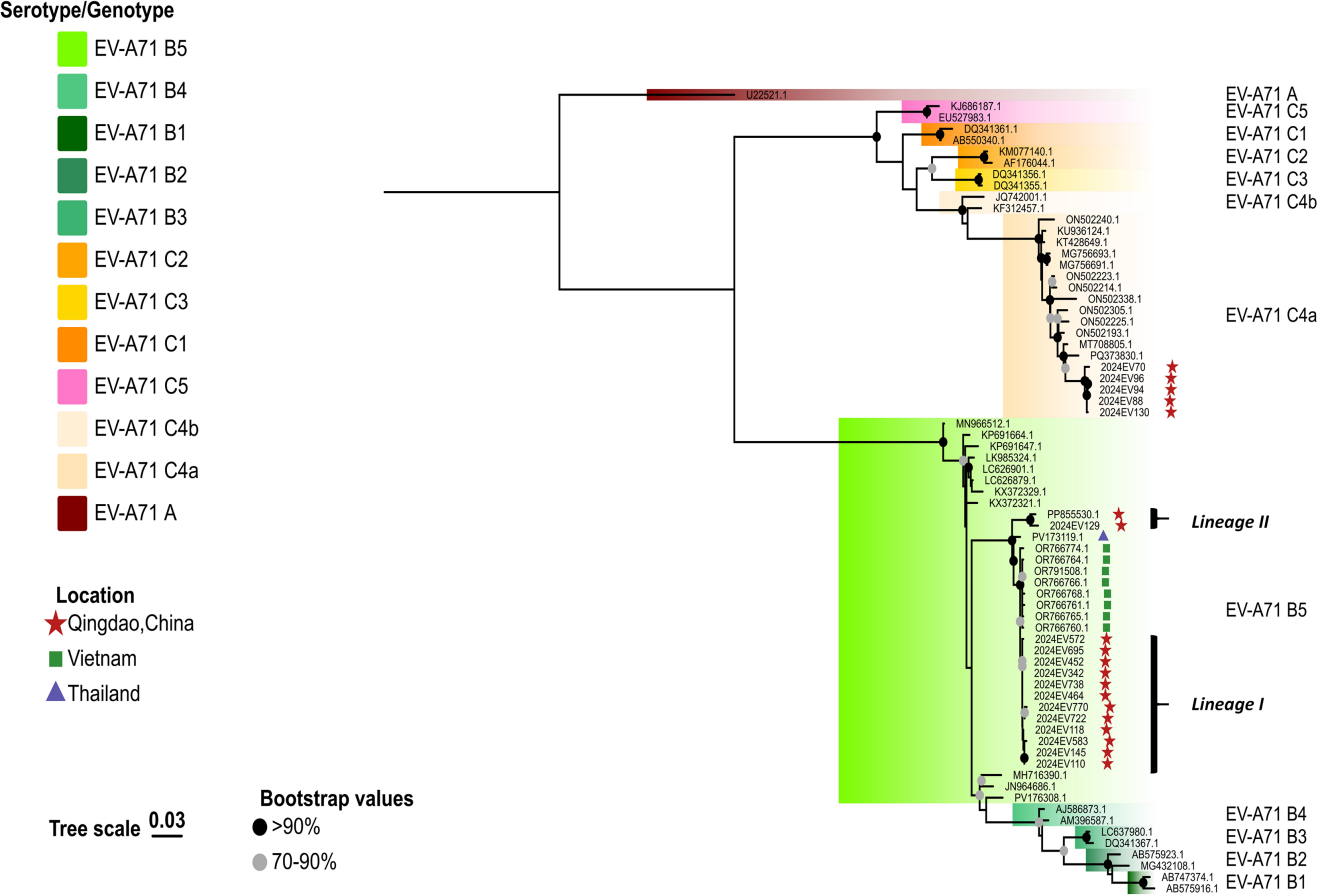
Phylogenetic analyses of the EV-A71 B5 strains were performed in this study. The phylogenetic tree was constructed using IQTREE with the maximum likelihood method based on full-length VP1 sequences. Bootstrap analysis was performed with 1000 replicates. Global representative EV-A71 sequences of each genotype were obtained from GenBank. Qingdao strains and selected representative strains were color-coded according to their respective genotypes or sub-genotypes. The scale bar indicates the ML of substitution per nucleotide position. Bootstrap values represent the percentage of 1,000 pseudoreplicate data sets.

**Figure 2:**
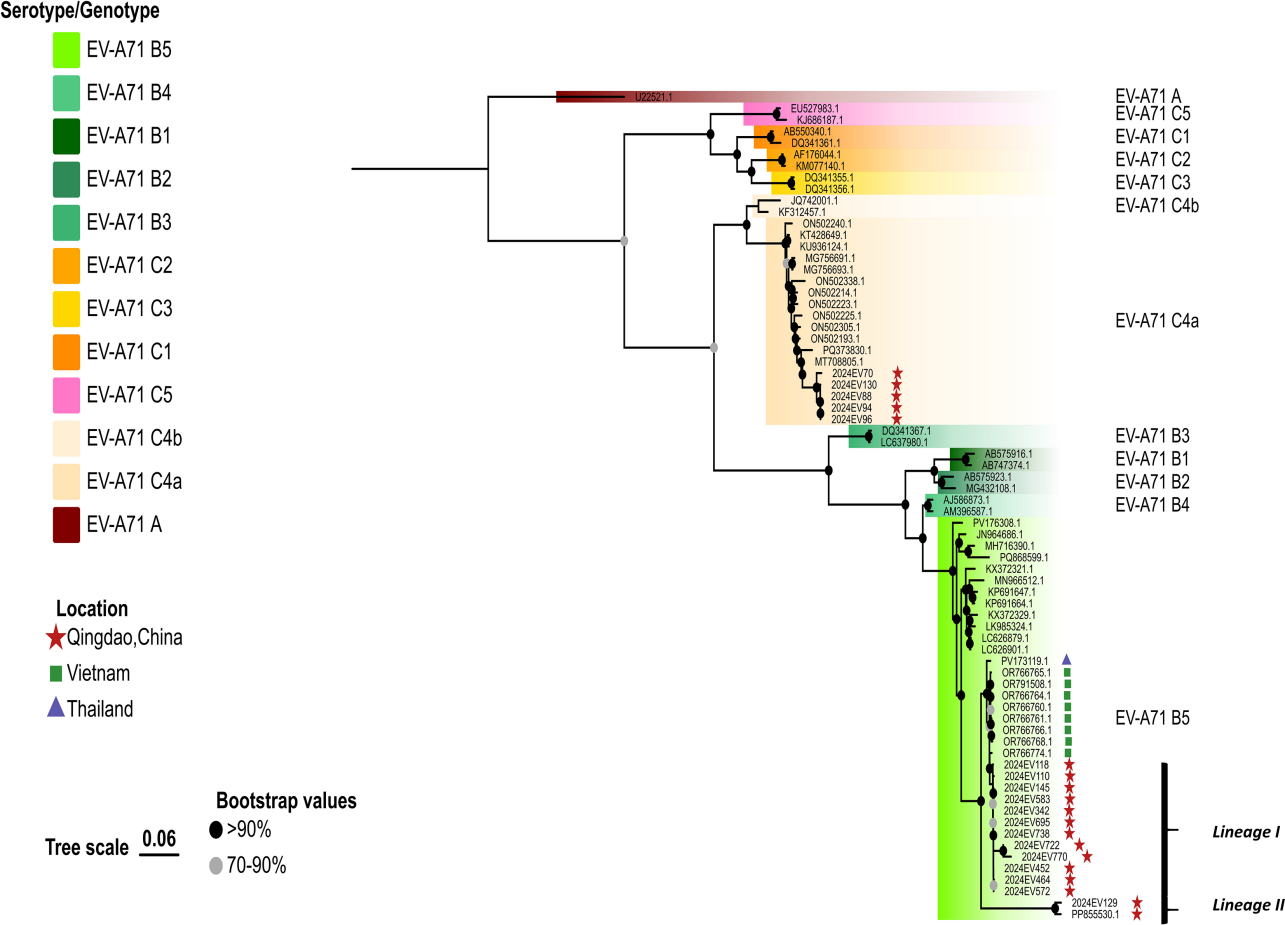
Phylogenetic analyses of the EV-A71 B5 strains were performed in this study. The phylogenetic tree was constructed using IQTREE with the maximum likelihood method based on complete genome sequences. Bootstrap analysis was performed with 1000 replicates. Global representative EV-A71 sequences for each genotype were obtained from GenBank. Qingdao strains and selected representative strains were color-coded according to their respective genotypes or sub-genotypes. The scale bar indicates the ML of substitution per nucleotide position. Bootstrap values represent the percentage of 1,000 pseudoreplicate data sets.

Phylogenetic analysis revealed that the five Qingdao EV-A71 C4 genotype strains formed a monophyletic clade exhibiting the closest genetic relationship to the Chinese reference strain 2019-EV-A71-R431 (GenBank accession number: MT708805.1) in both the VP1 and complete genome phylogenies (**Figures 1**, **2**). Compared to currently circulating EV-A71 C4 genotype strains with available genome sequences in NCBI, these five Qingdao strains contained multiple distinct lineage-specific mutations: two in the structural P1 region (VP1-V249I, VP3-T240S), two in the non-structural P2 region (2A-H25Y, 2B-A27T), and two in the non-structural P3 region (3B-K12R, 3D-R254K).

In contrast, all 14 EV-A71 B5 genotype isolates clustered into two distinct clades (designated Lineage I and Lineage II) in both VP1 and whole-genome phylogenies (**Figures 1**, **2**). Lineage I (n=12, 85.7%), which comprised the majority of isolates from 2024, was the predominant lineage. Within the VP1 phylogeny, these isolates exhibited closest genetic similarity to Vietnamese EV-A71 B5 genotype isolates from 2023 HFMD outbreaks. The whole-genome phylogeny confirmed this close relationship for ten Lineage I isolates. Conversely, two isolates (2024EV722 and 2024EV770) demonstrated significant genetic divergence from these Vietnamese references (**Figure 2**). Lineage II consisted of two variants: one collected in 2023 (GenBank accession number: PP855530.1) and the other in 2024. These variants demonstrated significant genetic divergence from Lineage I on both trees (**Figure 1**, **Figure 2**).

Analysis against Vietnamese EV-A71 B5 strains revealed conserved serine at VP1 position 17 in all Qingdao EV-A71 B5 Lineage I strains, while Vietnamese isolates exhibited an S17G (serine → glycine) substitution (**Supplementary Table S6).**

**Figure 3:**
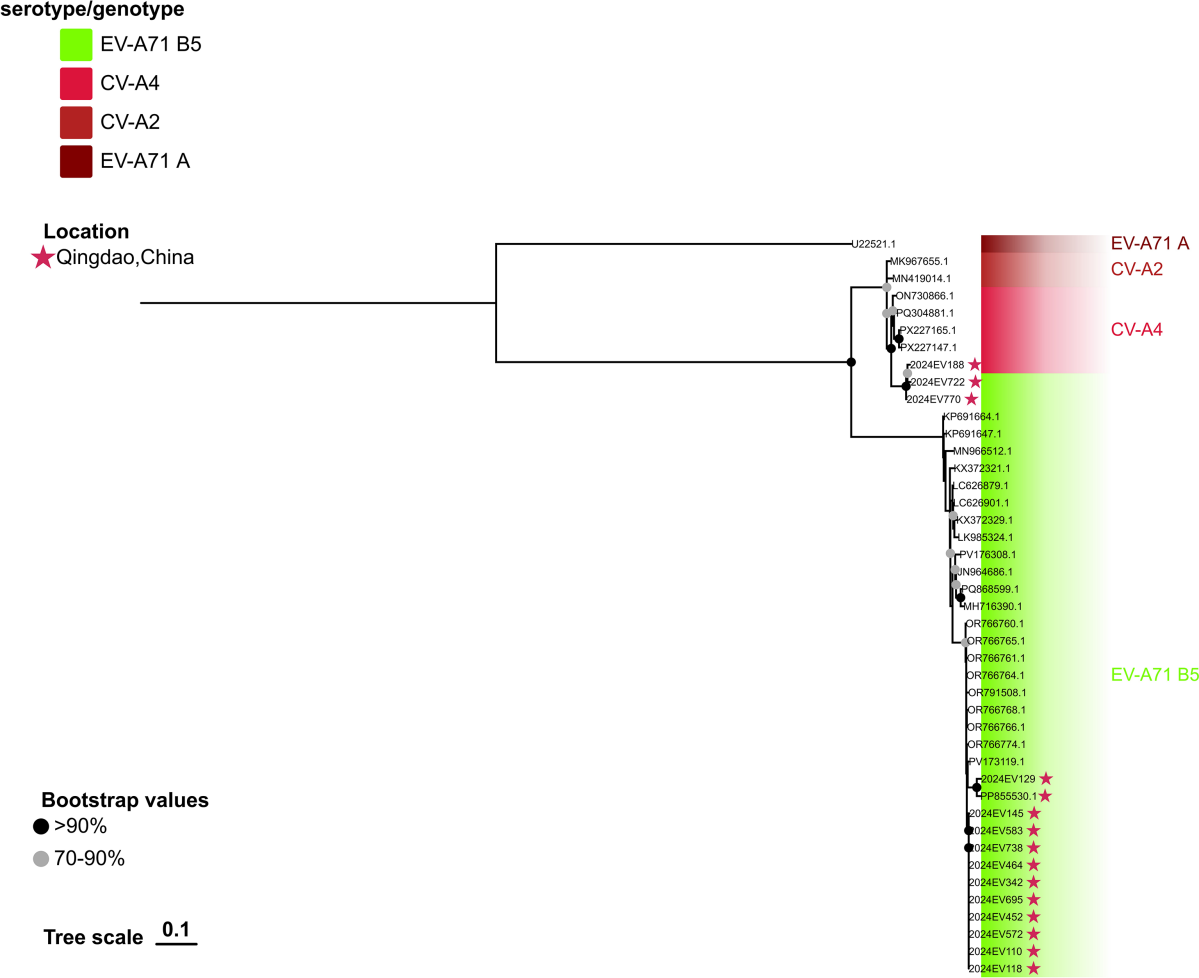
Phylogenetic analyses of the EV-A71 B5 strains and the CV-A4 strain were performed in this study. The phylogenetic tree was constructed using IQTREE with the maximum likelihood method, based on the partial 2C region (nucleotide positions 4,494 to 5,110; 617 bp). Bootstrap analysis was performed with 1,000 replicates. The scale bar indicates the ML of substitution per nucleotide position. Bootstrap values represent the percentage of 1,000 pseudoreplicate data sets.

**Figure 4:**
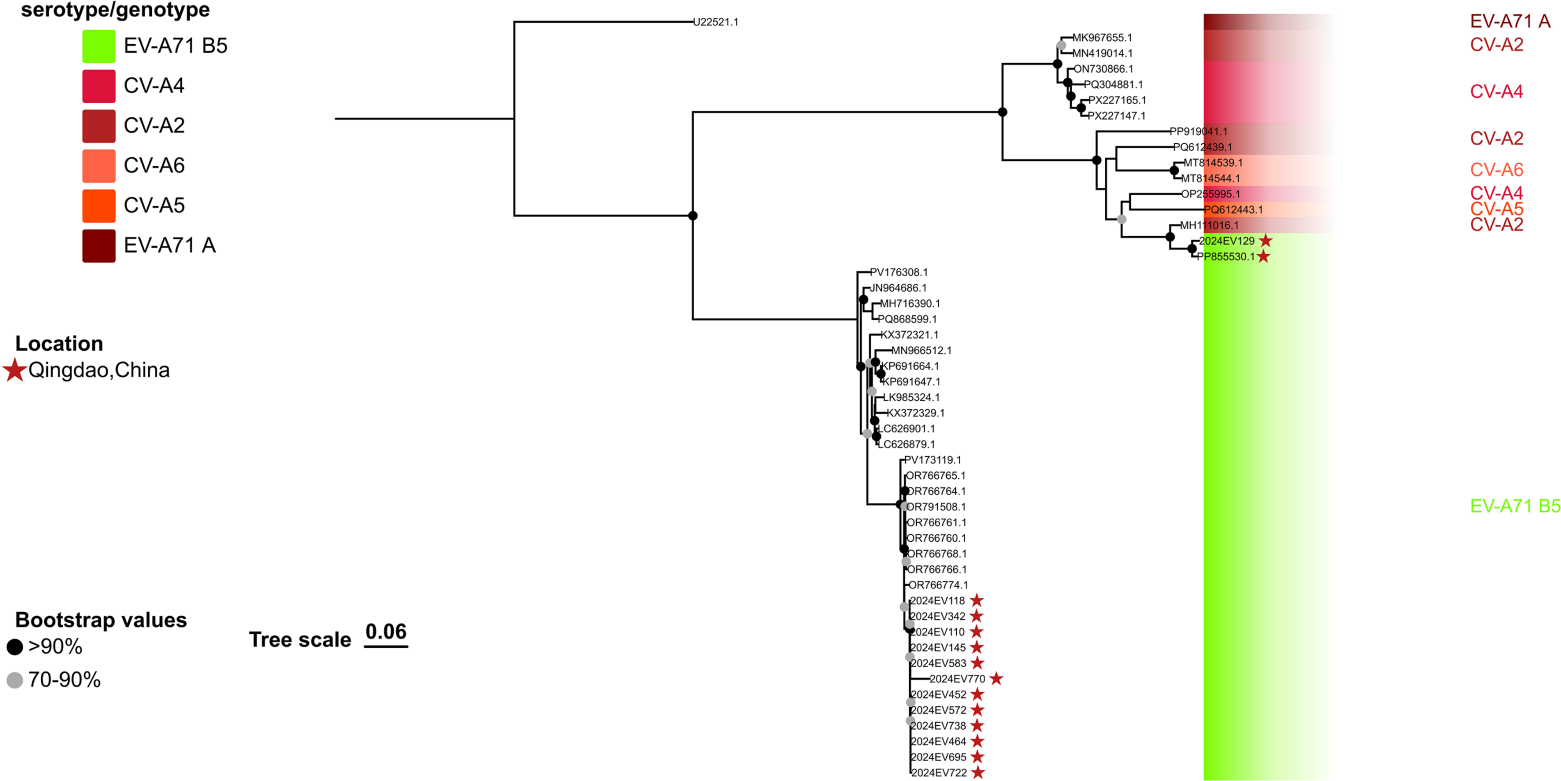
Phylogenetic analyses of the EV-A71 B5 strains and the CV-A4 strain were performed in this study. The phylogenetic tree was constructed using IQTREE with the maximum likelihood method, based on the partial 2C and 3C joint region (nucleotide positions 4,522 to 5,492; 971 bp). Bootstrap analysis was performed with 1,000 replicates. The scale bar indicates the ML of substtution per nucleotide position. Bootstrap values represent the percentage of 1,000 pseudoreplicate data sets.

Detailed genome analysis further characterized the divergent Lineage I B5 isolates. Isolate 2024EV722 exhibited high sequence identity with a contemporaneous Qingdao 2024 CV-A4 isolate (2024EV188) within the 2C region (nucleotides 4,494 to 5,110; 617 bp) (**Figure 3**). Similarly, isolate 2024EV770 displayed the highest sequence identity with the same CV-A4 strain across the 2C to 3C region (nucleotides 4,522 to 5,492; 971 bp) (**Figure 4**). These findings suggest that both isolates likely represent recombinant viruses.

**Figure 5:**
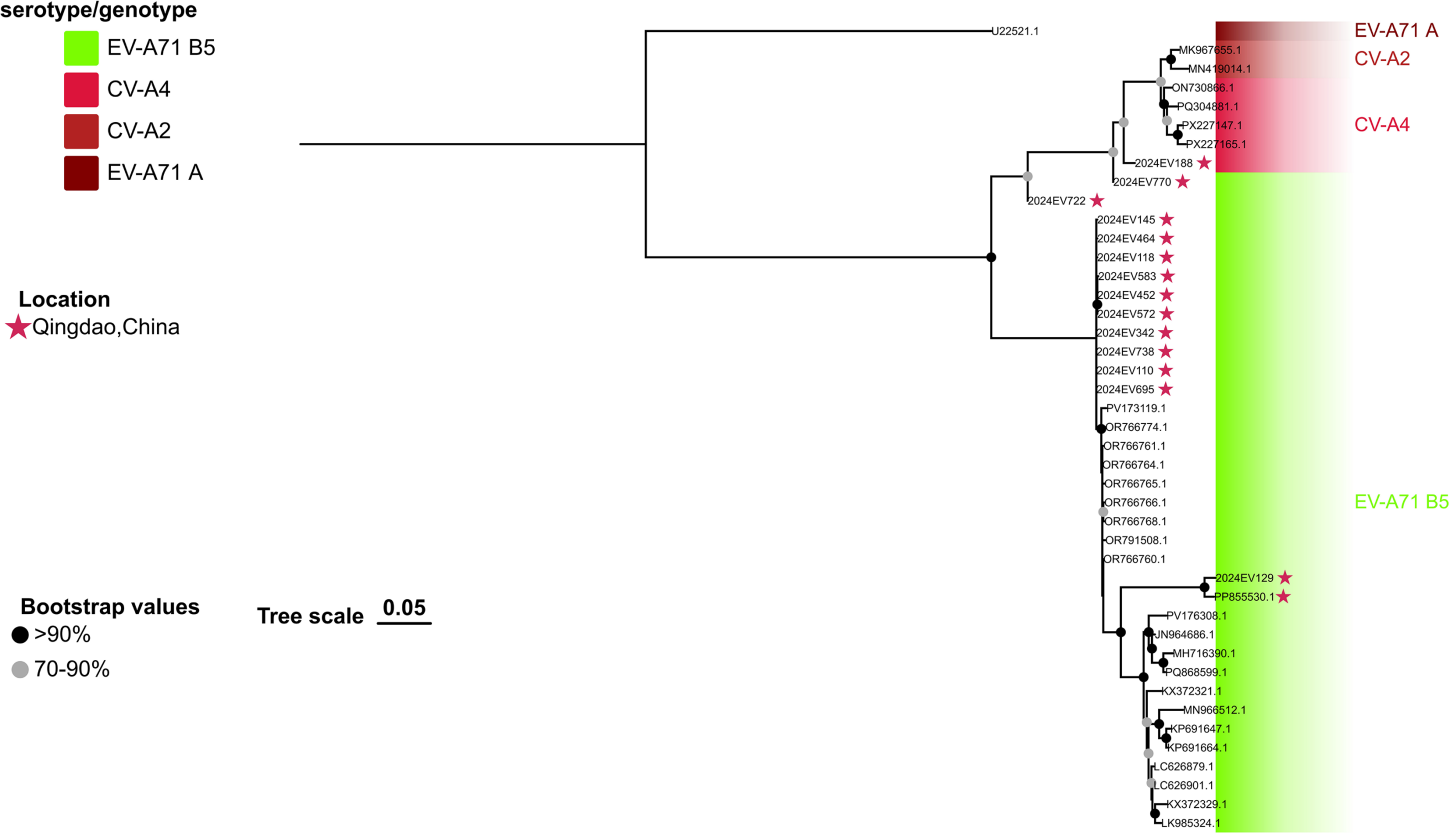
Phylogenetic analyses of the EV-A71 B5 strains and the CV-A2 strain were performed in this study. The phylogenetic tree was constructed using IQTREE with the maximum likelihood method based on the 3A-3D region (approximately spanning nucleotides 5,118 to 7,440; 2,323 bp). Bootstrap analysis was performed with 1,000 replicates. The scale bar indicates the ML of substitution per nucleotide position. Bootstrap values represent the percentage of 1,000 pseudoreplicate data sets.

Lineage II consisted of two isolates: one collected in 2023 (GenBank accession number: PP855530.1) and the other in 2024. Both viruses exhibited strict conservation of the VP1 S17 residue and shared the highest sequence identity with a 2023 Thailand EV-A71 B5 genotype isolate (GenBank accession number: PV173119.1) in the P1 and P2 regions. However, they displayed distinct substitution patterns unique to this lineage. These variations, which were shared among the Lineage II strains, consisted of clusters of synonymous and non-synonymous nucleotide substitutions across the structural (P1) and non-structural (P2) regions. The two lineage II-specific variations were marked by clusters of synonymous and non-synonymous nucleotide substitutions across the structural (P1) and non-structural (P2) regions. Notably, the non-structural P3 region exhibited disproportionately high and nonlinear sequence divergence. Within the 3A-3D region (approximately between nucleotides 5,118 and 7,440; 2,323 bp), two isolates shared highest identity with a 2016 Australian CV-A2 isolate (GenBank accession number: MH111016.1; **Figure 5**). This pronounced genome-wide evolutionary divergence indicates that Lineage II has followed a distinct evolutionary trajectory compared to Lineage I, suggesting that Lineage II isolates represent recombinant viruses. Furthermore, Lineage II isolates were detected across consecutive epidemic seasons (2023–2024), indicating continuous circulation. Collectively, these unique evolutionary patterns require further investigation.

### Recombination analysis

Since the P2 and P3 regions of the EV-A71 sequences in this study were analyzed individually using BLAST, it was found that 2024EV722 and 2024EV770 exhibit the strongest recombination signals with a contemporaneously obtained CV-A4 genome sequence (2024EV188) from Qingdao; PP855530.1 and 2024EV129 exhibit the strongest recombination signals with a CV-A2 genome sequence (MH111016.1, Australia, 2016).

Recombination analysis using SimPlot v3.5.1 revealed distinct recombination patterns consistent with the above-mentioned phylogenetic reconstruction for strains 2024EV722, 2024EV770, PP855530.1, and 2024EV129, supported by bootstrap values >70%. Analytical details are summarized in **Supplementary Figure S2**.

Complementary validation using RDP4 (v4.101) supported the recombination profiles, which were consistent with those obtained from SimPlot analysis. The four B5 genotype viruses exhibited significant recombination signals (p< 0.001) detected by all seven algorithms (RDP, GENECONV, BootScan, MaxChi, Chimaera, SiScan, and 3Seq). Regarding lineage I strains (2024EV722 and 2024EV770), both strains shared parental sequences, with the major parent identified as the other lineage I EV-A71 B5 genotype viruses, and the minor parent as the CV-A4 isolate 2024EV188 (GenBank accession: PV684192), collected in Qingdao during the present study. Notably, the recombination architectures differed between strains:2024EV722 exhibited recombination within the 2C region (between nucleotides 4,494–5,110; 617 bp, **Table 1**), whereas 2024EV770 showed an extended recombination spanning the 2C-3C region (between nucleotides 4,522–5,492; 971 bp, **Table 1**). For lineage II strains (PP855530.1 and 2024EV129), consensus recombination signals were detected across all algorithms with strong statistical support (p < 0.001). The recombinant block (approximately between nucleotides 5,118–7,440; 2323 bp) was mapped to the P3 region, but the actual breakpoint position remains undetermined, likely due to overprinting by a subsequent recombination event. Parental assignment identified PV173119.1 (EV-A71 B5 genotype, Thailand, 2023) as the major donor and MH111016.1 (CV-A2, Australia, 2016) as the minor donor, suggesting intertypic recombination between distinct enterovirus serotypes (**Table 1**).

**Table 1:** Recombination event screening of EV-A71 genotype B5 strains by RDP4 software.

Strains	Breakpoints		Parental sequence		Detection methods (p-value)						
	Beginning	Ending	Major	Minor	RDP	GENECONV	BootScan	Maxchi	Chimaera	SiScan	3Seq
PP855530.1	5118	7440*	PV173119.1	MH111016.1	4.491×10^-155^	1.381×10^-132^	7.050×10^-130^	2.422×10^-45^	3.084×10^-48^	4.717×10^-49^	3.730×10^-14^
2024EV129	5118	7440*	PV173119.1	MH111016.1	4.491×10^-155^	1.381×10^-132^	7.050×10^-130^	2.422×10^-45^	3.084×10^-48^	4.717×10^-49^	3.730×10^-14^
2024EV770	4522	5492	2024EV583	2024EV188	2.001×10^-135^	1.325×10^-118^	2.844×10^-133^	8.524×10^-30^	5.011×10^-29^	5.863×10^-37^	5.551×10^-15^
2024EV722	4494	5110	2024EV583	2024EV188	1.368×10^-97^	2.272×10^-86^	1.049×10^-96^	7.509×10^-21^	3.563×10^-20^	1.361×10^-25^	4.663×10^-14^

*The exact breakpoint position is undetermined, as it was likely overwritten by a subsequent recombination event. 2024EV583, a representative virus from lineage I EV-A71 B5, underwent recombination with CV-A4.Discussion.

## Discussion

Since the introduction of inactivated EV-A71 vaccines in China in 2016, national surveillance has documented a substantial decline in EV-A71 transmission (Head et al., 2020; Jiang et al., 2021; Hong et al., 2022; Wu et al., 2022; Xiao et al., 2022b; Zheng et al., 2023), a trend that has also been observed in Qingdao (**Supplementary Table S6**). Prior to 2023, the EV-A71 C4 genotype remained persistently dominant across China, with B5 genotype isolations largely confined to sporadic travel-associated cases in southern provinces (Yang et al., 2016; Huang et al., 2020; Chen et al., 2021). Notably, no EV-A71 B5 strains had been identified in Qingdao since surveillance began in 2007. However, during the 2023–2024 surveillance period, an unexpected epidemiological shift occurred: from the exclusive dominance of C4 to C4/B5 co-circulation.

In this study, consistent age and gender distributions, as well as overall enterovirus positive rates, between the two years-findings confirmed by this retrospective analysis rule out potential confounding factors (e.g., changes in population susceptibility or detection bias), supporting the conclusion that the observed increase reflects genuine EV-A71 circulation rather than methodological artifacts. Furthermore, EV-A71 transmission was sporadic, characterized by the absence of clustered outbreaks, broad geographic distribution across 10 districts, and heterogeneous case onset times; these features substantiate that the increase represents genuine community-wide spread rather than localized amplification. Collectively, the stable demographic and virological profiles, coupled with a diffuse spatiotemporal pattern, indicate that the observed rise in EV-A71 detection rates among HFMD cases in Qingdao (from 0.17% in 2023 to 2.73% in 2024; χ² = 24.28, p < 0.001) constitutes a genuine epidemiological trend.

Phylogenetic analysis revealed co-circulation of two genotypes-5 C4 and 14 B5 isolates—marking the first evidence of local B5 transmission in Qingdao. This shift from C4 dominance to C4/B5 co-circulation underscores the dynamic evolution of EV-A71 in the region and highlights the need for continued molecular surveillance to assess potential lineage-specific transmission advantages.

Genetic analysis showed that the five novel C4 strains formed a distinct monophyletic clade most closely related to the Chinese reference strain MT708805.1 (2019-EV-A71-R431), indicating sustained local diversification of the C4 genotype in China. Structurally, VP1—the primary determinant of virus neutralization—encompasses multiple antigenic sites (Yuan et al., 2018), including residue position 249, previously identified as under positive selection (Xiao et al., 2022a). Nonstructural proteins 2A, 2B, and 3B contribute to viral replication and immune evasion (Yuan et al., 2018). While 3D acts as the RNA-dependent RNA polymerase, driving genome replication and modulating host inflammatory and immune responses (Yuan et al., 2018; Nie et al., 2024). Compared with MT708805.1, all Qingdao C4 isolates carried the VP1-V249I substitution, though the functional impact of this substitution remains unclear. Additionally, identical lineage-specific substitutions in 2A, 2B, 3B, and 3D were shared among all Qingdao C4 isolates, warranting further investigation into their synergistic effects on viral fitness. Notably, comparative genomic analysis indicated that the C4 variants identified in this study were more closely related to the reference strain MT708805.1 than to the C4 variants obtained from GenBank that circulated from 2020 to 2023. This pattern suggests the possibility of cryptic transmission or surveillance gaps, highlighting the importance of longitudinal monitoring to assess the epidemic potential of this C4 lineage.

In contrast, the B5 genotype diverged into two distinct independent lineages, reflecting diverse transmission origins. Lineage I, characterized by the conserved VP1 amino acid S17, phylogenetically clustered with 2023 Vietnamese outbreak-associated strains, suggesting potential cross-border transmission. Lineage II constituted a basal clade with no definitive epidemiological links, indicating either potential cryptic spillover or recombination-driven evolutionary processes (subsequently corroborated by recombination analysis). Importantly, all Qingdao B5 strains maintained the conserved VP1 amino acid residue S17—in contrast to the S17G substitution predominantly fixed in severe cases from Vietnam—a finding that may explain the lack of severe disease among Qingdao cases and offers novel epidemiological evidence supporting a role for VP1 S17 in modulating viral pathogenicity (Chau et al., 2024). Geographically, the 2023 Vietnamese outbreak was defined by the co-circulation of the S17 lineage and the predominant S17G lineage, whereas no S17G variants were identified among Qingdao B5 strains. Potential explanations for this observed discrepancy include cryptic local circulation of S17G variants, the lack of imported S17G variants, or suboptimal local adaptation of such variants. These hypotheses warrant validation via expanded epidemiological surveillance.

Recombination, a well-established driver of enteroviral genetic diversity (Lukashev, 2005; Simmonds and Welch, 2006; Huang et al., 2019; Xu et al., 2021), serves a pivotal role in modulating viral virulence, antigenicity, and outbreak potential. Growing evidence implicates recombination of nonstructural gene segments during co-circulation in the emergence of novel EV-A71 lineages and sustained outbreaks (Hu et al., 2011; Noisumdaeng et al., 2018; Nikolaidis et al., 2019), as illustrated by the 2008 Fuyang outbreak (Zhang et al., 2010) and recent epidemics (Bible et al., 2008; Huang et al., 2009; Noisumdaeng et al., 2018; Xiao et al., 2022a), which aligns with the observations in the present study. Three recombinant B5 lineages identified in the present study displayed heterogeneous recombination profiles, reflecting frequent interspecies genetic recombination between EV-A71 and co-circulating enteroviruses (CV-A4, CV-A2). Lineage I strains (2024EV722 and 2024EV770) constituted genomic chimeras originating from Qingdao B5 strains and co-circulating CV-A4 strains, with recombination breakpoints mapping to the 2C and 2C-3C junction regions, respectively. In contrast, Lineage II variants (2024EV129/PP855530.1) acquired nearly the entire functionally divergent P3 region (approximately between nucleotides 5,118–7,440) through modular genomic exchange with CV-A2. The ancestral origins of these segments were phylogeographically disparate. Marked spatiotemporal divergence among these parental strains indicates that enteroviruses sustain genetic diversity over extended periods via cryptic transmission networks, wherein interregional and temporal recombination provides genetic substrates for adaptive evolution. Continuous detection of recombinant strains between 2023 and 2024 underscores their epidemic potential, emphasizing the importance of systematic, long-term epidemiological surveillance to evaluate their impact on HFMD epidemiological trends.

In conclusion, while this study is limited by the absence of complete EV-A71 sequences from certain years and geographic regions, based on the available data, this work enhances our understanding of the geographical distribution of EV-A71 by confirming the sustained circulation of genotype B5 in northern China and identifying a novel C4/B5 co-circulation pattern in Qingdao—a pattern reflecting complex local evolutionary dynamics. These two B5 lineages display divergent evolutionary trajectories: Lineage I, associated with Vietnamese strains and maintaining the conserved VP1 S17 residue, exhibits interregional transmission potential; Lineage II, a unique recombination-derived clade, suggests adaptation to distinct selective pressures. Sustained C4 diversification and key amino acid variations further provide novel insights into viral adaptive evolution. These findings have three key implications: first, they challenge the traditional assumption of B5 geographic restriction to southern China; second, they identify recombination and key variations as critical targets for investigating viral pathogenic mechanisms; third, they emphasize the need to expand genomic sequencing coverage to track the transmission of B5 and recombinant strains, thereby refining surveillance strategies and guiding HFMD prevention and control measures. Future research should incorporate larger sample sizes, long-term time-series surveillance, and *in vitro* functional assays to elucidate the biological significance of key variations and recombination events.

## Data Availability

The datasets presented in this study can be found in online repositories. The names of the repository/repositories and accession number(s) can be found below: https://www.ncbi.nlm.nih.gov/, PV684174-PV684192.
