# Supplementary material for: Genetic characterization of human enterovirus A71 genotypes C4 and B5 Circulating in Qingdao City, Shandong province, China, from 2023 to 2024
Source: Front Cell Infect Microbiol. 2025 Dec 16;15:1684067. doi: 10.3389/fcimb.2025.1684067 (PMC12748185; doi:10.3389/fcimb.2025.1684067)
Supplement: Supplementary file 1 [file DataSheet1.pdf]

Genetic Characterization of Human Enterovirus A71 genotypes C4 and B5 Circulating in Qingdao City, Shandong Province, China, from 2023 to 2024

Jinling Gong[1]*, Sitong Liu[1]*, Song Liu[1]*, Rui Sun[1], Siying Xiao[1], Zhilei Su[1], Xin Jiang[1], Qiuge Zhang[3], Xiaoyan Shi[1#], Xianming Liu[2#], Zhaoguo Wang[1#]

1. Qingdao Municipal Center for Disease Control and Prevention, Qingdao, Shandong, People's Republic of China. 2. Department of Neurosurgery, Qingdao Municipal Hospital, Qingdao, China. 3. Department of Epidemiology and Health Statistics, The College of Public Health of Qingdao University, Qingdao, China.

*: co-first author: Jinling Gong(gongjinling1@126.com), Sitong Liu(745130893@qq.com), Song Liu(liusongrr@163.com).

#: co-corresponding authors: Zhaoguo Wang(wzg-003@163.com), Xianming Liu(liuxm716@126.com), Xiaoyan Shi(didima@126.com)

author: Rui Sun(sunrui8608@163.com), Siying Xiao(49924655@qq.com), Zhilei Su(448929667@qq.com), Xin Jiang(jx_21@sina.com), Qiuge Zhang(zqg0725@163.com)

Supplementary Table S1. Age Descriptive Statistics

	N	Mean	Min	Max	SD	SEM	Median
2023	1167	4.73	0.08	19	3.59	0.11	4
2024	916	4.67	0.08	16	2.59	0.09	4
Difference		0.06				0.02	
overall	2083	4.70			3.19	0.07	4

Supplementary Table S2. Age Two-sample independent T-test

	t Statistic	DF	Prob>|t|
Equal Variance Assumed	0.41593	2081	0.6775
Equal Variance NOT Assumed (Welch Correction)	0.43215	2066.27878	0.66568

Null Hypothesis: mean1-mean2 = 0

Alternative Hypothesis: mean1-mean2 <> 0

At 0.05 level, when equal variance is assumed, Mean1 - Mean2 is NOT significantly different from 0

At 0.05 level, when equal variance is NOT assumed, Mean1 - Mean2 is NOT significantly different from 0

Supplementary Table S3. Age Confidence Intervals for mean

Conf. Levels in %	Lower Limits	Upper Limits
95	-0.21751	0.33461

Supplementary Table S4. Gender Statistics

	Female	Meal	Total
2023	404	763	1167
2024	341	575	916
Total	745	1338	2083

Supplementary Table S5. Gender Chi-Square Test

	Chi-Square	DF	Prob > ChiSq
Pearson Chi-Square	1.5663	1	0.21075
Likelihood Ratio	1.56451	1	0.21101
Continuity Correction	1.45314	1	0.22803

According to Pearson Chi-Square test:

At the 0.05 level, there is NOT significant evidence of association between two variables.

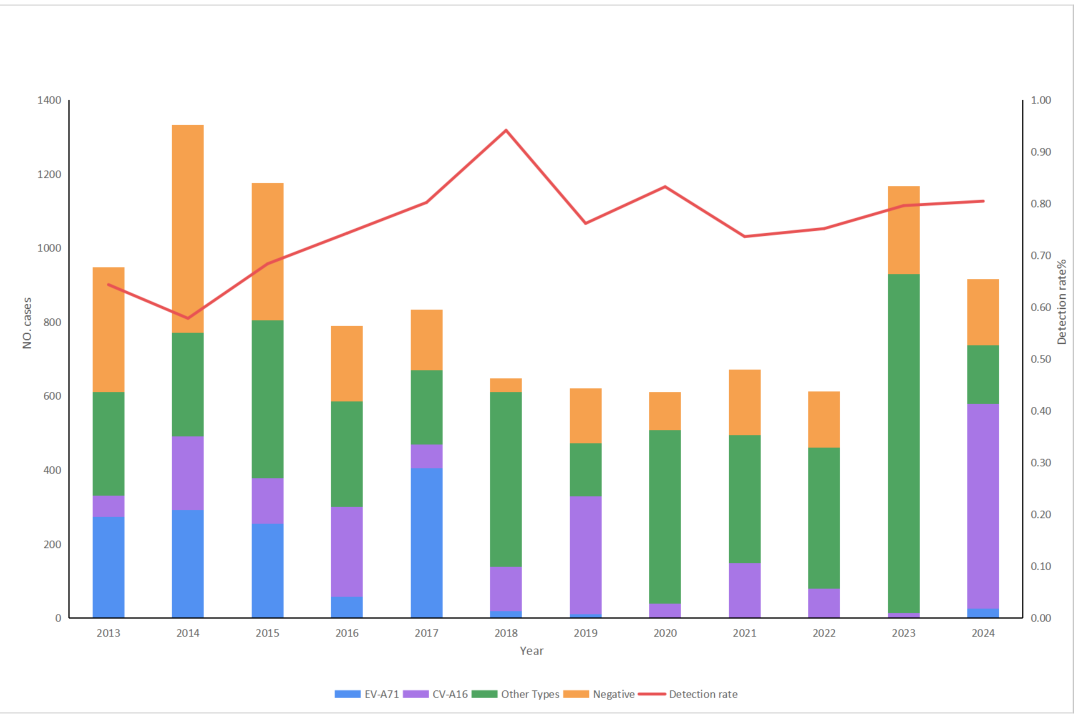

Supplementary Figure S1. The pathogen spectrum of HFMD in Qingdao from 2013 to 2024.

Supplementary Table S6. Amino acid sequence comparison of VP1 of the 14 EV-A71 B5 subgenotype viruses in Qingdao

**Position of Amino Acids on the EV-A71 VP1**	**1**	**2**	**3**	**4**	**5**	**6**	**7**	**8**	**9**	**10**	**11**	**12**	**13**	**14**	**15**	**16**	**17**	**18**	**19**	**20**
Strain No.																				
2024EV110	G	D	R	V	A	D	V	I	E	S	S	I	G	D	S	V	S	R	A	L
2024EV118	G	D	R	V	A	D	V	I	E	S	S	I	G	D	S	V	S	R	A	L
2024EV342	G	D	R	V	A	D	V	I	E	S	S	I	G	D	S	V	S	R	A	L
2024EV452	G	D	R	V	A	D	V	I	E	S	S	I	G	D	S	V	S	R	A	L
2024EV464	G	D	R	V	A	D	V	I	E	S	S	I	G	D	S	V	S	R	A	L
2024EV572	G	D	R	V	A	D	V	I	E	S	S	I	G	D	S	V	S	R	A	L
2024EV695	G	D	R	V	A	D	V	I	E	S	S	I	G	D	S	V	S	R	A	L
2024EV145	G	D	R	V	A	D	V	I	E	S	S	I	G	D	S	V	S	R	A	L
2024EV583	G	D	R	V	A	D	V	I	E	S	S	I	G	D	S	V	S	R	A	L
2024EV738	G	D	R	V	A	D	V	I	E	S	S	I	G	D	S	V	S	R	A	L
2024EV722	G	D	R	V	A	D	V	I	E	S	S	I	G	D	S	V	S	R	A	L
2024EV770	G	D	R	V	A	D	V	I	E	S	S	I	G	D	S	V	S	R	A	L
2024EV129	G	D	R	V	A	D	V	I	E	S	S	I	G	D	S	V	S	R	A	L
PP855530.1	G	D	R	V	A	D	V	I	E	S	S	I	G	D	S	V	S	R	A	L
OR766766.1	G	D	R	V	A	D	V	I	E	S	S	I	G	D	S	V	G	R	A	L
OR791508.1	G	D	R	V	A	D	V	I	E	S	S	I	G	D	S	V	G	R	A	L
OR766764.1	G	D	R	V	A	D	V	I	E	S	S	I	G	D	S	V	G	R	A	L
OR766760.1	G	D	R	V	A	D	V	I	E	S	S	I	G	D	S	V	G	R	A	L

OR766765.1	G	D	R	V	A	D	V	I	E	S	S	I	G	D	S	V	G	R	A	L
OR766768.1	G	D	R	V	A	D	V	I	E	S	S	I	G	D	S	V	G	R	A	L
OR766761.1	G	D	R	V	A	D	V	I	E	S	S	I	G	D	S	V	G	R	A	L
OR766774.1	G	D	R	V	A	D	V	I	E	S	S	I	G	D	S	V	S	R	A	L

Note: The whole genome sequence of an EV-A71 B5 subgenotype strain collected in Qingdao in 2023 is available under GenBank accession number PP855530.1. Additionally, eighteen whole genome sequences of EV-A71 strains and one of a CV-A4 strain, all collected in Qingdao in 2024, have been deposited in GenBank under accession numbers PV684174-PV68419.

Query:2024EV129(B5)

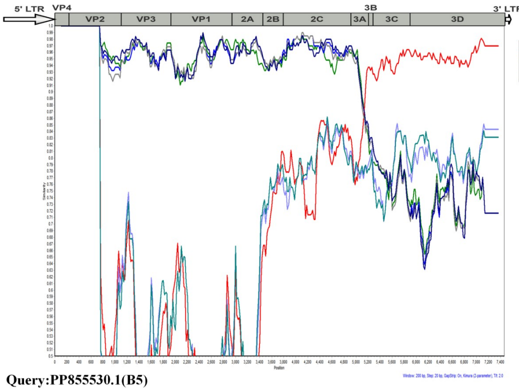

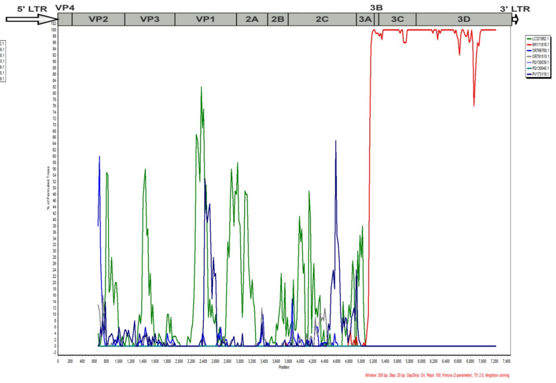

Query:PP855530.1(B5)

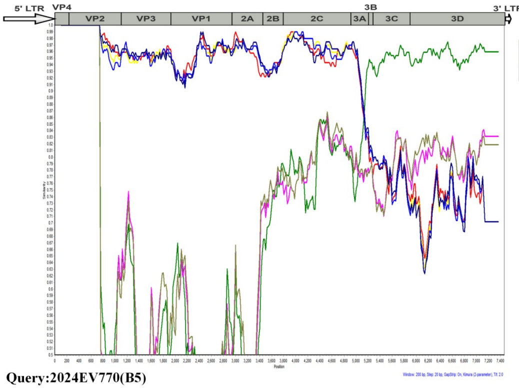

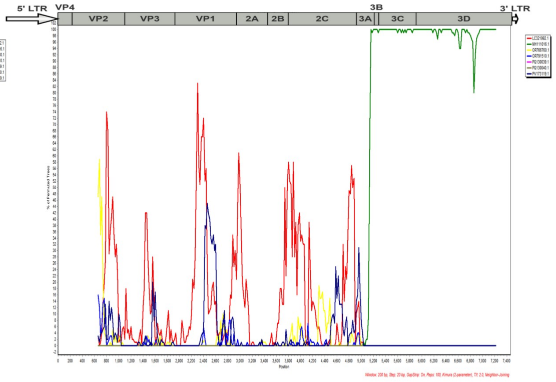

Query:2024EV770(B5)

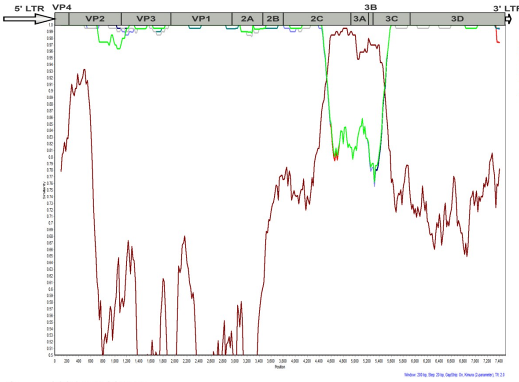

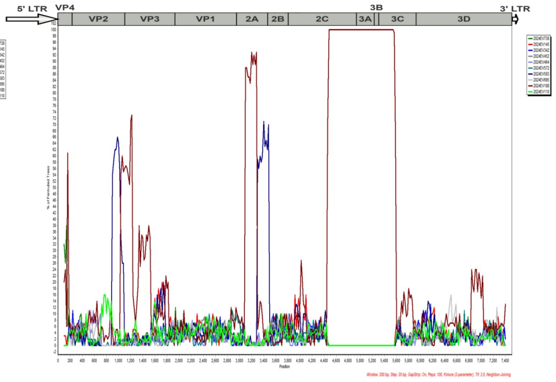

Query:2024EV722(B5)

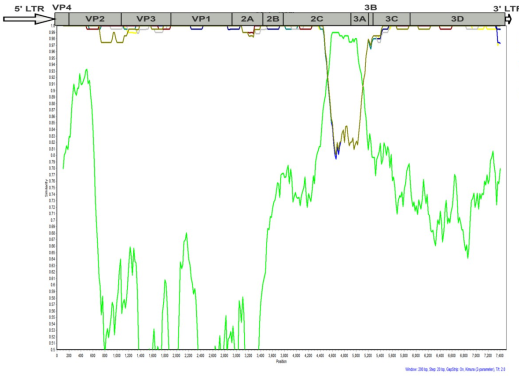

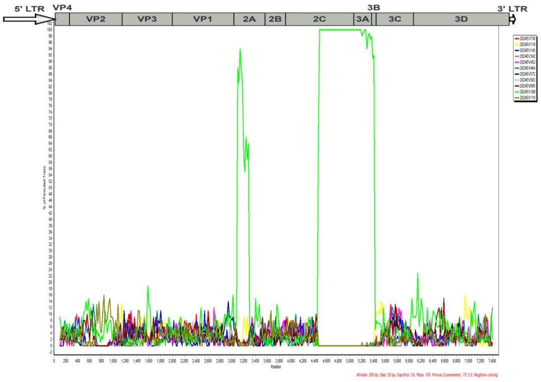

Supplementary Figure S2. Similarity plots and bootscan analyses of the four EV71 B5 genotype strains based on complete genomes using SimPlot. The graph on the left column shows the similarity plots, the graph on the right column figure shows the bootscan analyses. The similarity was calculated in a sliding window size of 200 nucleotides using the Kimura 2-parameter distance method. Bootscan analysis was performed using the

neighbor-joining tree model and the Kimura 2-parameter distance algorithm with a window size of 200 nucleotides.
